# p63 drives invasion in keratinocytes expressing HPV16 E6/E7 genes through regulation of Src-FAK signalling

**DOI:** 10.18632/oncotarget.3892

**Published:** 2015-05-07

**Authors:** Kirtiman Srivastava, Adam Pickard, Simon McDade, Dennis J McCance

**Affiliations:** ^1^ Centre for Cancer Research and Cell Biology, Queen's University Belfast, Belfast BT9 7BL, UK; ^2^ Department of Pathology, School of Medicine, University of New Mexico, Albuquerque, NM 87131-0001, USA

**Keywords:** HPV16, Src, p63, MMP14, invasion

## Abstract

Using microarray information from oro-pharyngeal data sets and results from primary human foreskin keratinocytes (HFK) expressing Human Papilloma Virus (HPV)-16 E6/E7 proteins, we show that p63 expression regulates signalling molecules which initiate cell migration such as Src and focal adhesion kinase (FAK) and induce invasion in 3D-organotypic rafts; a phenotype that can be reversed by depletion of p63. Knockdown of Src or FAK in the invasive cells restored focal adhesion protein paxillin at cell periphery and impaired the cell migration. In addition, specific inhibition of FAK (PF573228) or Src (dasatinib) activities mitigated invasion and attenuated the expression/activity of matrix metalloproteinase 14 (MMP14), a pivotal MMP in the MMP activation cascade. Expression of constitutively active Src in non-invasive HFK expressing E6/E7 proteins upregulated the activity of c-Jun and MMP14, and induced invasion in rafts. Depletion of Src, FAK or AKT in the invasive cells normalised the expression/activity of c-Jun and MMP14, thus implicating the Src-FAK/AKT/AP-1 signalling in MMP14-mediated extra-cellular matrix remodelling. Up-regulation of Src, AP-1, MMP14 and p63 expression was confirmed in oro-pharyngeal cancer. Since p63 transcriptionally regulated expression of many of the genes in this signalling pathway, it suggests that it has a central role in cancer progression.

## INTRODUCTION

Cases of head and neck squamous cell carcinomas (HNSCC) are increasing globally by 600,000/annum. A significant proportion of HNSCC are oro-pharyngeal cancers and at least 25% of these cancers are associated with the Human Papilloma Virus (HPV) infection, in particular HPV-16, which also causes cervical cancer [1, 2]. Oro-pharyngeal cancer patients with HPV infection respond better to the available therapies than the HPV-negative indicating a potential diversity in these cancers. The oncogenic transformation of HPV-positive tumours is believed to be triggered by integration of viral genome into the host chromosome, leading to upregulated expression of E6 and E7 proteins which are well known to degrade p53 and retinoblastoma proteins, respectively. The E6 and E7 proteins transcriptionally regulate many cellular genes including the p63 family, which are overexpressed in oro-pharyngeal cancers [3, 4].

p63, a member of the p53 transcription factor family, is essential for development of stratified epithelium and limbs as inherited recessive mutations in the p63 gene cause cranial developmental problems with cleft palate, skin defects and hypoplasia of breasts in humans [5, 6]. Recent microarray studies have highlighted the oncogenic properties of certain p63 isoforms in inducing epithelial to mesenchymal transition, cell migration, extracellular matrix (ECM) remodelling and invasion [7–9].

Aberrant activities of proteins regulating cell motility can promote invasion. Genes from the non-receptor tyrosine kinase family i.e. protein tyrosine kinase 2 (PTK2; which encodes focal adhesion kinase (FAK)) and Src are known regulators of cell migration [10, 11]. Activation of FAK (Tyr^576/577^) and Src (Tyr^416^) are regulated by mutual phosphorylation events at the indicated amino acids near cell-matrix adhesion sites, leading to the formation of an activated Src-FAK complex. In addition, Src activity is also regulated by phosphorylation of the C-terminal negative regulatory Tyr^530^ site, as point mutations or loss of C-terminal residues results in constitutively active Src, which is often seen in colon cancers patients [12, 13]. Studies with tumour tissue arrays have shown a direct correlation between activated Src-FAK complex and increase in tumour progression [14–16]. The activated Src-FAK complex is known to mediate the phosphorylation of scaffold proteins like paxillin at Tyr^118^ to trigger the disassembly of focal contacts, which is prerequisite for cell migration [17].

Cancer cells also secrete increased amounts of proteinases like matrix metalloproteinases (MMPs), which degrade different components of ECM and basement membrane to facilitate the invasion [18]. MMPs are zinc-dependent endopeptidases which are activated through cleavage of their precursors either by proteases like plasmin or other active MMPs including collagenases (MMP1), gelatinases (MMP2, MMP9), stromelysins (MMP3) and membrane-type MMPs (MMP14). Most of these MMPs are overexpressed in most types of HNSCC and are linked with poor prognosis of the disease. Thus, the signalling pathways regulating the gene expression of these MMPs are critical in disease progression [19].

In the tumour microenvironment, the increased presence of inflammatory mediators stimulate the expression of MMPs through induction of either extracellular signal-regulated kinase (ERK), c-Jun NH_2_-terminal kinase (JNK) or phosphoinositide 3-kinase/AKT signalling pathways [19]. We have previously shown that AKT, a serine/threonine kinase, regulates epithelial cell migration and invasion [20, 21]. Despite these findings, the underlying molecular mechanisms driving epithelial invasion after HPV16 infection still remains obscure, and is the focus of this study.

Here, we show that p63, Src, PTK2, c-Jun (activator protein-1 (AP-1) transcription factor) and MMP14 genes are upregulated in patients with oro-pharyngeal cancers. Using human foreskin keratinocytes (HFK) expressing HPV16 E6 and E7 genes (E6/E7-HFK) we show that increased expression of p63 promotes migration and invasion in a Src-FAK-dependent mechanism. We further identified Src as pivotal regulator of ECM remodelling via AKT/AP-1/MMP14 dependent pathway.

## RESULTS

### p63 expression is required for invasion of late passage E6/E7-HFK

**Figure 1:**
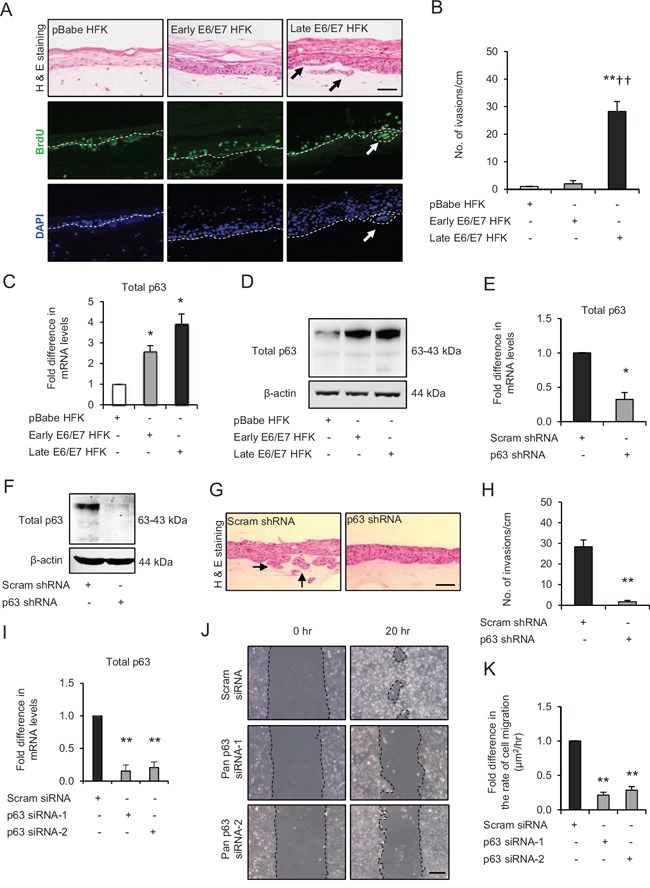
p63 transcription factors drive cell migration and invasion in late passage human foreskin keratinocytes (HFK) expressing human papilloma virus (HPV)16 E6/E7 genes **A**. H&E staining and immunofluorescence detection of BrdU uptake and nuclei (DAPI) on 3D-organotypic rafts established from normal HFK expressing pBabe (control), early passage and late passage HFK expressing E6/E7 genes cells seeded on retinoblastoma-depleted human foreskin fibroblast embedded collagen-I plugs. Invasive incidents are indicated by arrows. **B**. Quantification of number of invasive incidents across the rafts per cm. **C**. The relative mRNA and **D**. protein expression of total p63 and loading control β-actin in aforementioned cells. **E**. Relative mRNA and **F**. protein levels of total p63 in late passage E6/E7-HFK after stable p63 (p63 shRNA) knockdown compared to its controls (scram shRNA). **G**. H&E staining of rafts established from late passage HFK expressing control and stable p63 knockdown. **H**. Quantification of number of invasive incidents per cm across the rafts. **I**. Relative mRNA levels of total p63 after transient knockdown by control and two different p63 siRNA molecules in late passage E6/E7-HFK. **J**. Representative phase contrast images of scratch wound assay, showing cell migration after 20 hr in these cells seeded on collagen-I coated plates. **K**. Quantification of migration represented as μm^2^/hr. Scale bars represent 100 μm. *N* = 3 independent experiments, mean ± SEM, **p < 0.01 compared to the respective controls, *p < 0.05 compared to the control, ^††^*p* < 0.01 compared to early passage E6/E7-HFK.

To investigate the role of p63 in oro-pharyngeal squamous cell cancers (OPSCC) we utilised transformed HFKs expressing high risk HPV16 E6 and E7 genes, which we have previously shown to invade into the collagen-I plugs when cultured with retinoblastoma-depleted fibroblasts [20, 22]. Importantly, comparison of control (pBabe) HFK with early and late passage E6/E7-HFK indicates that invasion only occurs in late passage cells (> 12 passages) (Fig. 1A and 1B), which is independent of cell proliferation as confirmed by similar BrdU incorporation in E6/E7-HFK (Supplementary Fig. 1A). Furthermore, mitomycin-C (inhibitor of cell proliferation)-treated late passage cells demonstrated higher rate of cell migration (*p* < 0.05) compared to the mitomycin-C-treated control and early passage E6/E7-HFK (Supplementary Fig. 2A and 2B) and indicated cell migration and not proliferation is essential for invasion.

**Figure 2:**
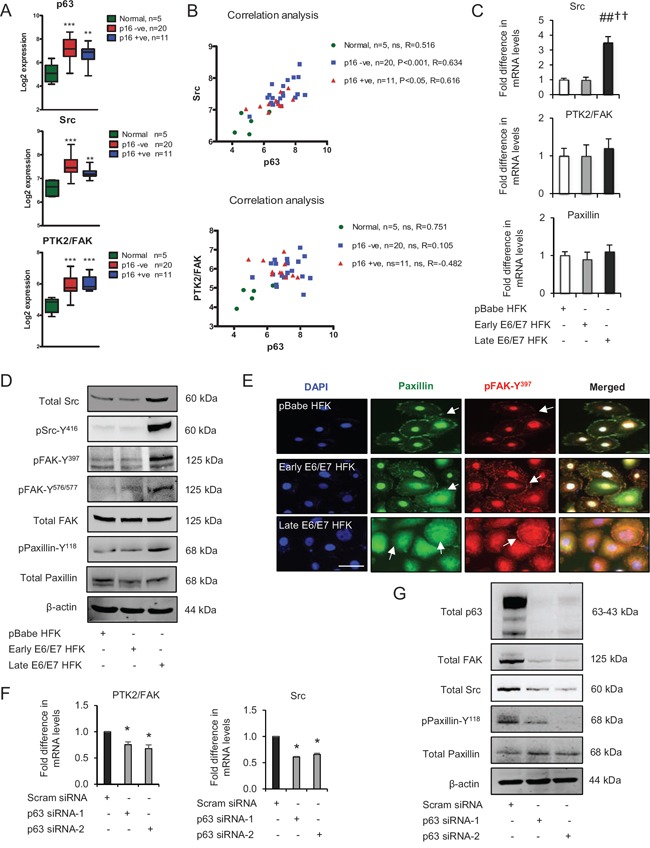
p63 transcription factors modulate Src-focal adhesion kinase (FAK) signalling **A**. Microarray data of total p63, Src and PTK2/FAK mRNA levels in normal tissue and HPV-negative (p16-) and HPV-positive (p16+) oro-pharyngeal tumours. **B**. Correlation analysis of Src and PTK2/FAK against p63 in samples from (A). **C**. Relative mRNA levels of Src, PTK2/FAK and paxillin in human foreskin keratinocytes (HFK) expressing pBabe (control), early and late passage HFK expressing E6/E7 genes. **D**. Protein levels of total Src, FAK, paxillin, pSrc-Y^416^, pFAK-Y^397^, pFAK-Y^576/577^ and pPaxillin-Y^118^; **E**. cellular localisation (arrows) of total paxillin and pFAK-Y^397^ in aforementioned cells. **F**. Relative mRNA levels of PTK2/FAK and Src in p63 depleted cells and **G**. protein levels of total p63, Src, FAK, paxillin and pPaxillin-Y^118^ after transient knockdown by Scram (control) and two different p63 siRNA molecules in late passage E6/E7-HFK. β-actin served as loading control. Scale bars represent 50 μm. *N* = 3 independent experiments, mean ± SEM, ***p < 0.0001 compared to normal, ^##^*p* < 0.01 compared to the control, *p < 0.01 compared to the control, ^††^*p* < 0.01 compared to early passage E6/E7 HFK, ns: not significant.

Analysis of mRNA (Fig. 1C) and protein (Fig. 1D) levels of p63 indicated that p63 is upregulated in cells expressing E6/E7 genes and further increased in late passage population. Stable shRNA mediated depletion of p63 (Fig. 1E and 1F) in late passage E6/E7-HFK resulted in a significant decrease in the invasive incidents (Fig. 1G and 1H) suggesting that p63 expression is pivotal for invasion. In addition, transient knockdown of p63 isoforms by two different siRNA molecules (Fig. 2G) in late passage E6/E7-HFK impaired the cell migration suggesting that p63 is required for a migratory phenotype (Fig. 1I–1K).

### p63 transcription factors modulate cell migration via Src-FAK signalling

Comparison of our previous genome wide ChIP-seq analyses of p63 function [9, 23] with microarray analyses of OPSCC [24] identified a number of p63 target genes whose expression like that of p63 (Fig. 2A) was elevated in both HPV positive, as measured by p16^INK4A^ [p16+], and HPV negative [p16-]) oro-pharyngeal cancers compared to normal tissue [23]. In particular, we observed that both HPV-positive and -negative OPSCC tumours had elevated levels of Src and PTK2 (encodes focal adhesion kinase (FAK)) genes (Fig. 2A), which were associated with p63 binding sites detected by ChIP-seq in normal cells [23]. Importantly, only the Src expression correlated significantly with p63 levels in OPSCC tumours (Fig. 2B). In addition, the mRNA and protein levels of total Src were higher in late passage E6/E7-HFK compared to the other cell populations, albeit total FAK and paxillin levels were relatively unchanged (Fig. 2C and 2D). Src levels correlated with a marked increase in the activation of the Src-FAK complex as represented by elevated levels of pFAK-Y^397^ (auto-phosphorylation), pSrc-Y^416^ and pFAK-Y^576/577^ resulting in enhanced levels of pPaxillin-Y^118^ which is a well-known target of the activated Src-FAK complex (Fig. 2D). In addition, late passage cells also appeared to have high rates of focal adhesion turnover, as suggested by increased cytoplasmic localisation of paxillin and pFAK-Y^397^ (Fig. 2E). In contrast, control HFK exhibited distinct peripheral localisation of paxillin, which coincided with faint peripheral localisation of pFAK-Y^397^. The early passage E6/E7-HFK exhibited an intermediary phenotype with punctate membrane staining of paxillin (Fig. 2E).

To confirm the correlation between p63 and Src-FAK signalling axis, the transient knockdown p63 isoforms by two different siRNA molecules in late passage E6/E7-HFK significantly reduced the mRNA and protein levels of PTK2 and Src and attenuated their activities as confirmed by depleted levels of pPaxillin-Y^118^ (Fig. 2F and 2G). These results indicate that p63 transcription factors regulate the expression and activities of FAK and Src signalling molecules, which may explain the impaired cell migration observed earlier after p63 knockdown.

### Src-FAK signalling regulates cell migration and invasion

**Figure 3:**
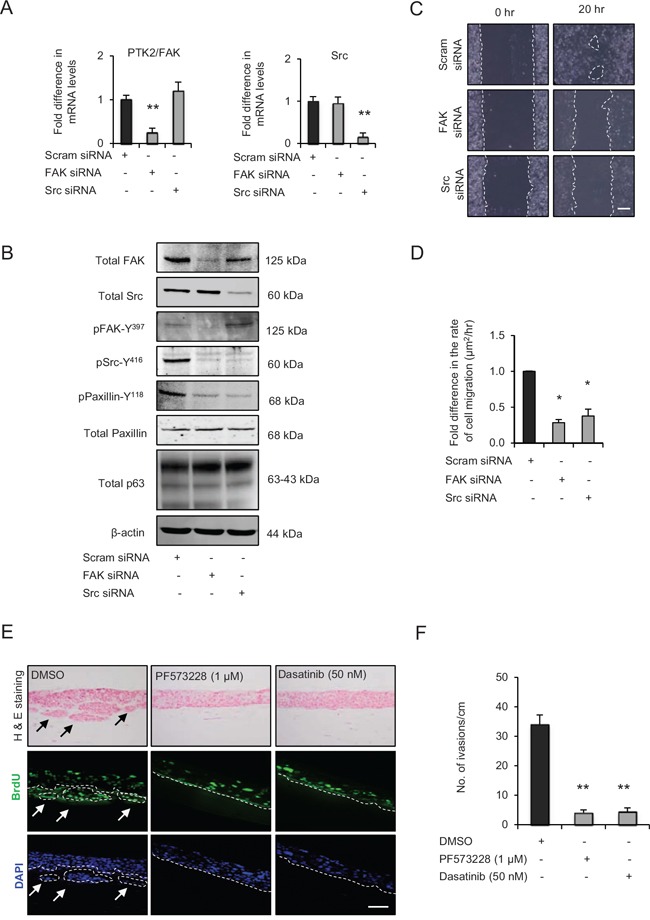
Src-focal adhesion kinase (FAK) signalling regulates cell migration and invasion **A**. Relative mRNA levels of PTK2/FAK and Src and, **B**. protein levels of total FAK, Src, paxillin, pSrc-Y^416^, pFAK-Y^397^, pPaxillin-Y^118^ and β-actin after transient knockdown by Scram (control), FAK and Src siRNA molecules in late passage human foreskin keratinocytes (HFK) expressing HPV16 E6/E7 genes. **C**. Representative phase contrast images of scratch wound assay on collagen-I coated plates showing cell migration after transient knockdown with scram, FAK and Src siRNA in the late passage E6/E7-HFK, which was **D**. quantified and represented as μm^2^/hr. **E**. H&E staining and immunofluorescence detection of BrdU uptake and nuclei (DAPI) on 3D-organotypic rafts established from late passage E6/E7-HFK seeded on retinoblastoma-depleted HFF embedded collagen-I plugs and treated with specific inhibitors of FAK (PF573228) and Src (dasatinib) activities. Rafts treated with DMSO served as control. Invasive incidents are indicated by arrows. **F**. Quantification of number of invasive incidents across the rafts per cm. Scale bars represents 100 μm. *N* = 3 independent experiments, mean ± SEM, **p < 0.01 compared to the respective controls, *p < 0.05 compared to the control.

To confirm the role of Src-FAK signalling axis in enhanced migration of late passage E6/E7-HFK, we depleted Src or FAK expression in these cells by transient siRNA knockdown (Fig. 3A and 3B). As expected since Src activity is in part dependent on FAK activity, the FAK or Src knockdown reduced pSrc-Y^416^ and pPaxillin-Y^118^ levels without altering the protein levels of total paxillin and p63 (Fig. 3B) and resulted in impaired cell migration compared to scrambled control (Fig. 3C and 3D) indicating that Src-FAK signalling enhanced cell migration through modulating the stability of focal adhesions. The reappearance of paxillin at cell periphery after FAK or Src knockdown confirms this notion and was similar to control cells (Supplementary Fig. 3 vs Figure 2E). Since p63 levels were unchanged in Src or FAK-depleted cells, these results indicate that p63 is not a transciptional target of Src-FAK signalling (Fig. 3B).

To ascertain the importance of FAK and Src in invasion and since we had used short acting siRNAs for the above experiments, we compared the effect of the specific inhibitors of FAK (PF573228) and Src (dasatinib) activities with control (DMSO), which resulted in significant decreases in the number of invasive incidents (Fig. 3E and 3F), with a minimal effect on cell proliferation (Supplementary Fig. 1B).

### MMP14-mediated ECM remodelling is pivotal for invasion

We have previously reported elevated levels of MMP1 and MMP14 in tissue sections from oro-pharyngeal cancer patients and also identified MMP14 as a direct p63 regulated gene in normal HFK [9, 22]. The microarray analysis of oro-pharyngeal cancers indicated increased levels of MMP1, but not MMP2, 9 and 14 in these tumours compared to normal tissue (Supplementary Fig. 4A) suggesting a potentially important role of MMPs in invasion.

**Figure 4:**
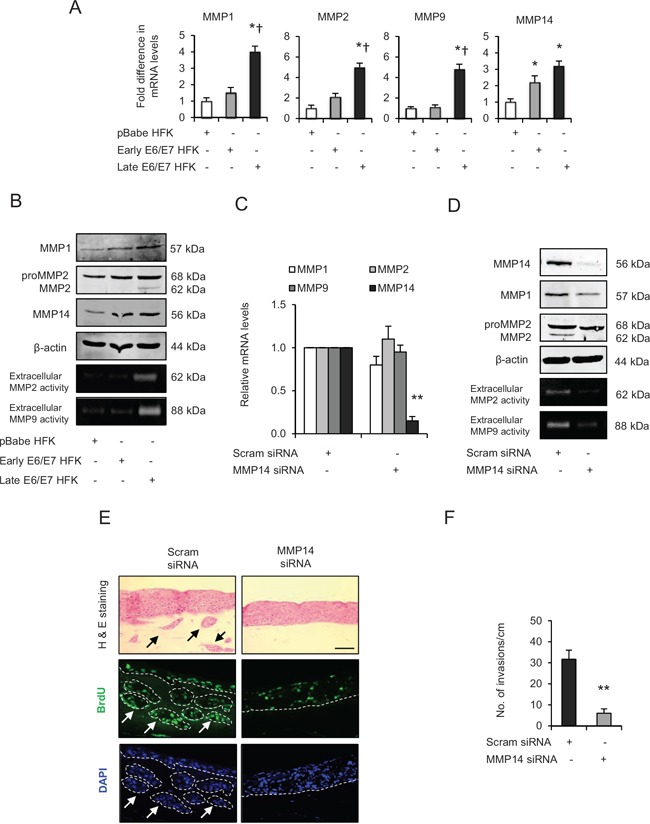
Matrix metalloproteinases (MMP)-mediated extra cellular matrix (ECM) remodelling is important during invasion **A**. The relative mRNA levels of MMP1, 2, 9 and 14, **B**. protein expression and activities on gelatin zymographs of MMPs in human foreskin keratinocytes (HFK) expressing pBabe (control), early and late passage HFK expressing HPV16 E6/E7 genes. **C**. Relative mRNA levels of MMP1, 2, 9 and 14; and **D**. protein levels of MMP1, 2, 14 and extracellular activities of MMP2 and MMP9 after transient knockdown by Scram (control) or MMP14 siRNA molecules in the late passage E6/E7-HFK. **E**. H&E staining and immunofluorescence detection of BrdU uptake and nuclei (DAPI) on 3D-organotypic rafts established from aforementioned MMP14 knockdown cells. Invasive incidents are indicated by arrows. **F**. Quantification of number of invasive incidents across the rafts per cm. β-actin served as loading control. Scale bar represent 100 μm. *N* = 3 independent experiments, mean ± SEM, **p < 0.01 compared to the control, *p < 0.05 compared to the controls, ^†^*p* < 0.05 compared to the early passage population.

The mRNA analysis indicated that levels of MMP1, 2 and 9 are upregulated (*p* < 0.05) in the late passage E6/E7-HFK compared to normal and early passage E6/E7-HFK (Fig. 4A), whereas, MMP14 levels were elevated (*p* < 0.05) even in early passage E6/E7-HFK compared to control and remained elevated in the late passage population (Fig. 4A). These results were supported by a gradual increase in protein levels of MMP1 and 2 from non-invasive to the invasive population (Fig. 4B). Importantly, while protein levels of MMP14 mirrored its mRNA expression, its enzymatic activity, as reported by the presence of cleaved MMP2 protein and extracellular MMP2-mediated digestion of gelatin, were elevated only in the invasive late passage E6/E7-HFK. This was also the case with MMP9 activity, which is potentially activated downstream of MMP2 (Fig. 4B).

Since MMP14 is well known to regulate the activities of MMP2 and MMP9 [25], we examined the effect of transient knockdown of MMP14 in late passage E6/E7-HFK (Fig. 4C and 4D). Depletion of MMP14 expression caused a marginal reduction in mRNA and protein levels of MMP1 while having no effect on the mRNA/protein expression of MMP2 and MMP9. However, the extracellular activities of MMP2 and MMP9 were dramatically reduced in the MMP14-knockdown cells (Fig. 4D). These results confirm the well known cascade of MMP activation and illustrate the importance of MMP14 [25]. Knockdown of MMP14, significantly attenuated the invasive incidents (Fig. 4E and 4F) without affecting cell proliferation (Supplementary Fig. 1C), thus highlighting the significance of MMP14 expression in driving invasion.

### Src-FAK signalling regulates MMP14-dependent ECM remodelling

**Figure 5:**
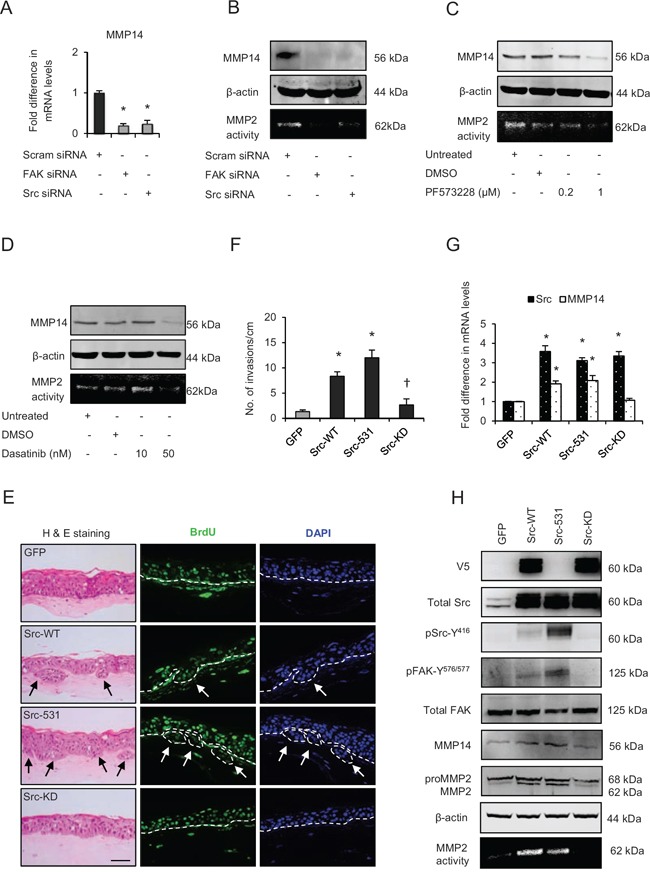
Src-focal adhesion kinase (FAK) signalling regulates expression and activity of matrix metalloproteinases-14 (MMP14) **A**. Relative mRNA levels of MMP14, **B**. protein levels and activity (observed by extracellular MMP2-mediated digestion of gelatin) of MMP14 after transient knockdown by Scram (control), FAK and Src siRNA molecules in late passage human foreskin keratinocytes (HFK) expressing HPV16 E6/E7 genes. **C and D**. Protein expression of total MMP14 and MMP14 activity in the late passage populations after dose-dependent inhibition of FAK (PF573228) and Src (dasatinib) activities. **E**. H&E staining and immunofluorescence detection of BrdU uptake and nuclei (DAPI) on 3D-organotypic rafts established from early passage HFK expressing E6/E7 genes alongside GFP (control), wild type-Src (Src-WT), constitutively active (Src-531) and kinase dead (Src-KD) constructs. The invasive incidents are indicated by arrows. **F**. Quantification of the number of invasive incidents across the rafts per cm. **G**. The relative mRNA levels of Src and MMP14 and **H**. protein levels of V5-tagged Src (Src-531 was not tagged with V5), total Src, pSrc-Y^416^, total FAK, pFAK-Y^576/577^, MMP2 and MMP14, and the activity of MMP14 in early passage HFK expressing E6/E7 genes and Src constructs. β-actin served as loading control. Scale bar represent 100 μm. *N* = 3 independent experiments, mean ± SEM, *p < 0.05 compared to the respective controls, ^†^*p* < 0.05 compared to the early passage population expressing Src-531.

Since specific inhibition of Src or FAK activities inhibited invasion, we next sought to determine their effect on the expression and activity of MMP14 since Src-signalling can regulate MMP levels [26]. Transient knockdown of FAK or Src (Fig. 5A) or specific inhibition of their activities appeared to deplete the mRNA and protein levels of MMP14 concomitant with reduction of the MMP2-mediated digestion of gelatin, thus confirming the attenuated MMP14 activity (Fig. 5B-5D). These results indicate that Src-FAK signalling may modulate the MMP14-mediated ECM remodelling by altering its expression and activity.

To further test this hypothesis we compared the effects of adenoviral-mediated wild type Src (Src-WT), constitutively active (Src-531) or kinase dead mutant (Src-KD) expression in early passage E6/E7-HFK, which do not invade. Our results indicate increased number of invasive incidents only in the Src-WT and Src-531 expressing cells (Fig. 5E and 5F), which was independent of proliferation as confirmed by similar BrdU uptake between all the groups (Supplementary Fig. 1D). Furthermore, the Src-WT and Src-531 expressing cells show elevated mRNA and protein levels of MMP14 alongside elevated levels of pSrc-Y^416^ and pFAK-Y^576/577^ which indicates the formation of activated Src-FAK complex and confirms that Src-mediated modulation of MMP14 expression and activity is pivotal for invasion (Fig. 5G and 5H). Depletion of Src or FAK did not affect p63 levels (Fig. 3B).

### AKT activity modulates MMP14 levels via an AP-1-dependent mechanism

**Figure 6:**
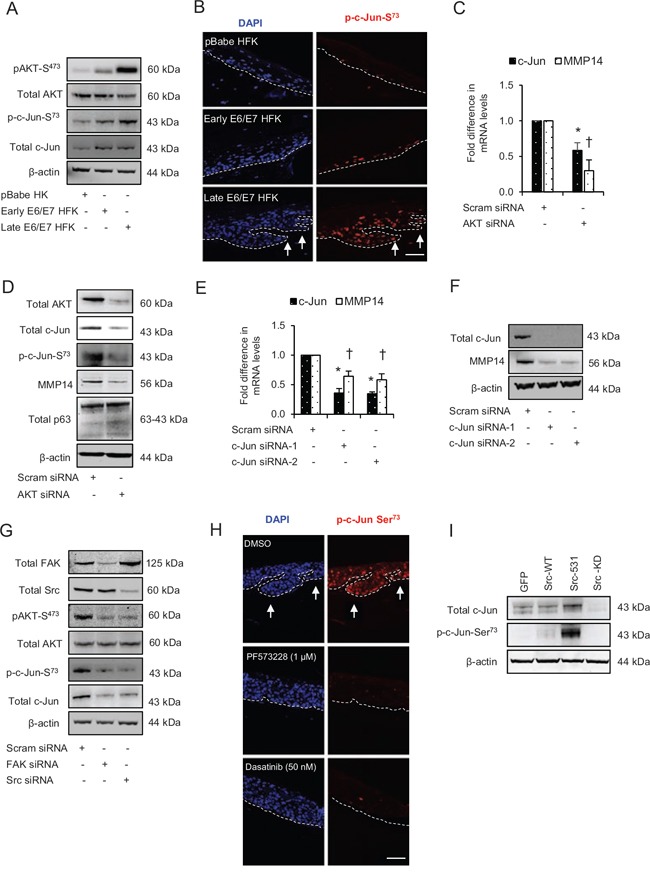
Src-focal adhesion kinase (FAK) signalling regulates the expression and activation of c-Jun via AKT activation **A**. The protein levels of pAKT-S^473^, total AKT, p-c-Jun-S^73^ and total c-Jun in the normal human foreskin keratinocytes (HFK) expressing pBabe (control), early passage and late passage HFK expressing HPV16 E6/E7 genes. **B**. Immunofluorescence detection of p-c-Jun-S^73^ on the rafts. **C**. The relative mRNA levels of c-Jun and MMP14, and **D**. protein levels of total AKT, total c-Jun, p-c-Jun-S^73^, MMP14 and total p63 after transient knockdown with scram (control) and total AKT siRNA in late passage E6/E7-HFK. **E**. The relative mRNA levels of c-Jun and MMP14, and **F**. protein levels of total c-Jun, MMP14 and β-actin after transient knockdown with scram (control) and two different c-Jun siRNA molecules in the late passage E6/E7-HFK. **G**. Protein levels of total FAK, total Src, pAKT-S^473^, total AKT, p-c-Jun-S^73^ and total c-Jun after transient knockdown by Scram (control), FAK or Src siRNA molecules in the late passage E6/E7-HFK. **H**. Immunofluorescence detections of p-c-Jun-S^73^ and BrdU uptake on 3D-organotypic rafts established from the late passage population and treated with specific inhibitors of FAK (PF573228) and Src (dasatinib) activities. Rafts treated with DMSO served as control. The invasive incidents are indicated by arrows. **I**. Protein levels of total c-Jun, p-c-Jun-S^73^ in early passage E6/E7-HFK expressing GFP, wild type-Src (Src-WT), constitutively active (Src-531) and kinase dead (Src-KD) constructs. β-actin served as loading control. Scale bars represent 100 μm. *N* = 3 independent experiments, mean ± SEM, *p < 0.05 compared to the respective controls, ^†^*p* < 0.05 compared to the respective controls.

We have previously shown that AKT signalling regulates cell migration and expression of MMPs [21, 22], so we investigated if AKT maybe downstream of the Src-FAK complex. There were marginal and dramatic increases in the levels of pAKT-S^473^ in early and late passage E6/E7-HFK, respectively, compared to control, although the total AKT levels were similar between the groups (Fig. 6A).

Since c-Jun, an AP-1 transcription factor, can be activated by AKT signalling pathway and it can regulate transcription of MMP14 [26, 27], we determined the expression and activity of c-Jun by analysing its total and serine^73^ phosphorylation level, respectively (Fig. 6A and 6B). This revealed that protein expression of c-Jun increased in both early and late passage E6/E7-HFK compared to the controls which was coupled with incremental increase in c-Jun activation as measured by p-c-Jun-S^73^ levels (Fig. 6A and 6B). Furthermore, the microarray analysis of OPSCC indicated increased levels of c-Jun in OPSCC compared to normal tissue (Supplementary Fig. 4B).

Therefore, to explore the link between AKT, c-Jun and MMP14, we compared effects of transient knockdown of total AKT in late passage E6/E7-HFK. Our results reveal significant decreases in the mRNA and protein levels of MMP14 and c-Jun following the knockdown of total AKT, which correlates with depletion of p-c-Jun^73^ (Fig. 6C and 6D). Since p63 levels were unchanged in AKT-depleted cells, these results indicate that AKT signalling may regulate MMP14 expression via AP-1-dependent mechanism. Indeed, the transient knockdown of c-Jun by two different siRNA molecules significantly reduced the mRNA and protein levels of MMP14 confirming this idea (Fig. 6E and 6F).

### Src-FAK signalling modulates AP-1 via AKT pathway

Since the activation of the Src-FAK complex drives invasion in E6/E7-HFK in part by regulating the expression and activity of MMP14, we explored the connection between Src signalling and AKT-dependent AP-1 activity. Transient knockdown of total FAK or Src proteins in the invasive population dramatically reduced pAKT-S^473^ levels without altering the total AKT levels, therefore suggesting the Src-FAK-mediated activation of AKT signalling pathway (Fig. 6G). The reduction in c-Jun and p-c-Jun-S^73^ levels supported our previous data following the depletion of AKT (Fig. 6D) as their protein levels were also depleted after Src or FAK knockdown (Fig. 6G). These results indicate that Src-FAK signalling modulates AKT activity, which in turn regulates the activity of AP-1 transcription factors. Indeed, specific inhibition of FAK and Src activities in rafts established with the invasive population showed a pronounced reduction in the nuclear staining of p-c-Jun-S^73^ compared to vehicle treated control (Fig. 6H) and a subsequent reduction in invasion as previous shown (Fig. 3E and 3F). In addition, the cells expressing the constitutively active Src-531 molecule demonstrated increased protein levels of c-Jun and p-c-Jun-S^73^, which confirms the Src-mediated AP-1 activation (Fig. 6I).

### p63 transcription factors modulate the activation of AKT/AP-1 signalling

**Figure 7:**
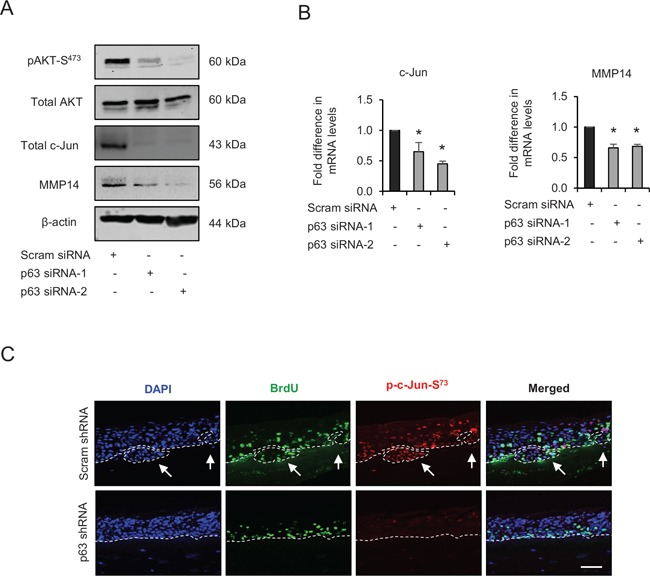
p63 regulates the expression and/or activation of AKT, c-Jun and matrix metalloproteinase-14 (MMP14) **A**. Protein levels of pAKT-S^473^, total AKT, total c-Jun, MMP14 and β-actin; and **B**. the relative mRNA levels of c-Jun and MMP14 after transient knockdown by Scram (control) and two different p63 siRNA molecules in late passage human foreskin keratinocytes (HFK) expressing human papilloma virus (HPV)16 E6/E7 genes. **C**. Immunofluorescence detections of p-c-Jun-S^73^ and BrdU uptake on 3D-organotypic rafts established from late passage HFK after stable p63 (p63 shRNA) knockdown compared to its control (scram shRNA). The invasive incidents are indicated by arrows. Scale bar represent 100 μm. *N* = 3 independent experiments, mean ± SEM, *p < 0.05 compared to the controls.

Our earlier results have shown a reduction in invasion after p63 knockdown. Since AKT activation is pivotal for cell migration and invasion, the role of p63 in activating AKT/AP-1 signalling was investigated. The transient knockdown of p63 by two different siRNA molecules in late passage E6/E7-HFK inhibited AKT activation as evident by diminished levels of pAKT-S^473^ although the total AKT levels remained unchanged (Fig. 7A). The p63 knockdown also reduced the mRNA and protein levels of c-Jun and MMP14, thus indicating that these proteins are potential transcriptional targets of p63 (Fig. 7A and 7B). In addition, rafts establish with late passage E6/E7-HFK expressing stable p63 knockdown demonstrated a striking disappearance of p-c-Jun-S^73^ from the nuclei (Fig. 7C). Since cell proliferation remained similar in the p63-depleted cells (Supplementary Fig. 1E), invasion is due to both p63-mediated regulation of AP-1 transcription and activity.

**Figure 8:**
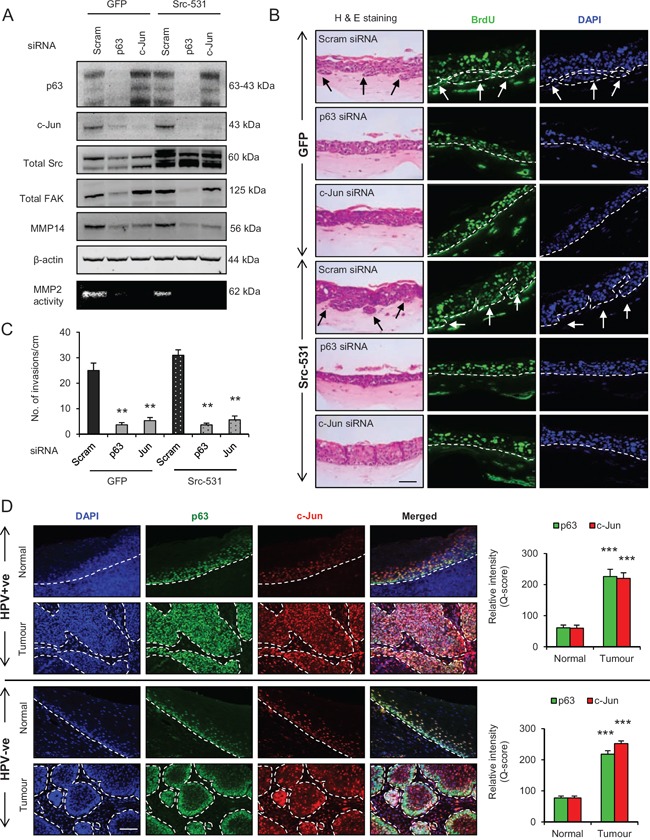
p63 regulates the Src/c-Jun/matrix metalloproteinases-14 (MMP14)-mediated extra cellular matrix (ECM) remodelling and invasion **A**. Protein levels of total p63, c-Jun, Src, focal adhesion kinase (FAK), MMP14 and β-actin; and activity of MMP2 as read out of the MMP14 activity after knockdown with Scram (control), total p63 or c-Jun siRNA in late passage human foreskin keratinocytes (HFK) expressing HPV16 E6/E7 gene alongside GFP or constitutively active Src (Src-531) constructs. **B**. H&E staining showing invasions (arrows) and immunofluorescence detection of BrdU uptake and nuclei (DAPI) on 3D-organotypic rafts established with cells from (A). **C**. Quantification of number of invasive incidents across the rafts per cm. **D**. Immunofluorescence detection and quantification (Q-score) of p63 and c-Jun in normal epithelium and tumour areas from HPV positive (+ve) and HPV negative (−ve) oro-pharyngeal squamous cell carcinomas. Scale bar represent 100 μm. *N* = 3 independent experiments, 5 (HPV+ve) and 5 (HPV−ve) tumour sections, mean ± SEM, ***p < 0.001 compared to the normal areas, **p < 0.01 compared to the controls.

Since p63 and c-Jun regulate expression of many genes, further studies were designed to confirm the link between these transcription factors and to study their significance in relation to invasion. Transient knockdown of p63 or c-Jun (Fig. 8A) in late passage E6/E7-HFK depleted the protein expression and activity of MMP14, and significantly reduced (*p* < 0.01) the invasive incidents on rafts (Fig. 8A-8C) with minimal effects on proliferation (Supplementary Fig. 1F). The same was true when we expressed the constitutively active Src molecule (Sc-531) in the p63 or c-Jun depleted late passage cells (Fig. 8A-8C), suggesting that p63 and c-Jun are required for invasion even in the presence of high levels of activated Src. Presumably this activity maintains levels of c-Jun and MMP14 for activation by Src-FAK/AKT. While the knockdown of p63 reduced c-Jun levels in the late passage E6/E7-HFK, the reverse was not observed which confirms that c-Jun is a target of p63 isoforms (Fig. 8A). These results emphasize the role of p63 as an upstream regulator of Src-FAK/AP-1 signalling through transcriptional regulation and activation.

To further validate our *in vitro* results from primary HFK expressing E6/E7 genes, oro-pharyngeal cancer sections with known HPV status (as confirmed by two independent methods i.e. p16 staining and chromogenic *in situ* hybridisation (CISH)) were used to study the expression and localisation of p63, c-Jun, Src and MMP14. In normal epidermis adjacent to either HPV positive or negative tumours, intense nuclear localisation of p63 was found only in the basal epithelial cells, with staining gradually fading in supra-basal epithelial population indicating differentiation and stratification of the normal epidermis (Fig. 8D). On the contrary, intense nuclear localisation of p63 was found throughout both HPV positive and negative tumours but was absent in the stromal cells. Similarly, the nuclear localisation of c-Jun appeared to be mainly in the basal and supra-basal regions of normal epithelium adjacent to both HPV positive and negative tissue and gradually disappeared due to stratification (Fig. 8D). However, pronounced nuclear localisation of c-Jun was observed in HPV positive and negative tumour areas and co-localised with p63 staining (Fig. 8D). Quantification of the level of increase in tumour tissue of both p63 and c-Jun is shown in Fig. 8D. In the stromal compartment, intense nuclear staining of c-Jun was only observed around the tumour areas of the sections, which may reflect the presence of enhanced paracrine signalling in the tumour microenvironment (Fig. 8D).

**Figure 9:**
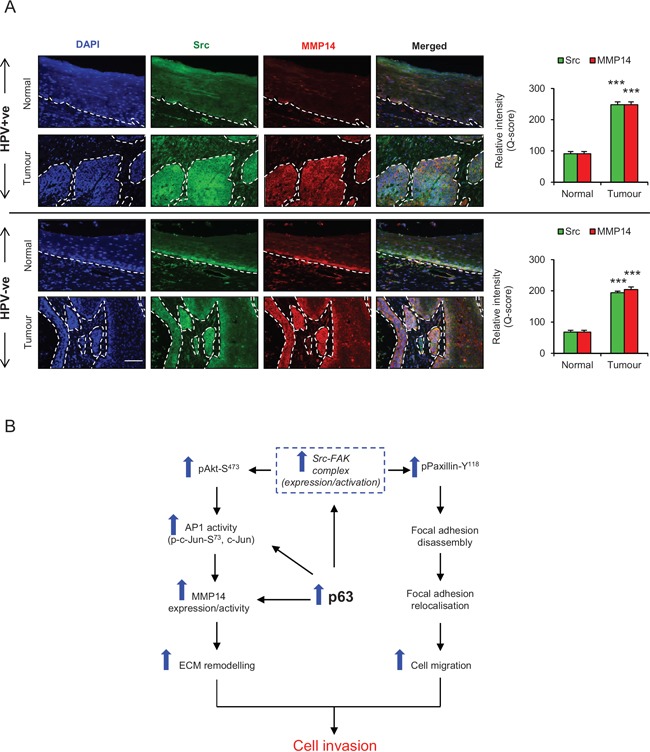
A. Immunofluorescence detection and quantification (Q-score) of Src and MMP14 in normal epithelium and tumour areas from HPV positive (+ve) and HPV negative (−ve) oro-pharyngeal squamous cell carcinomas The nuclei are stained with DAPI. **B**. Schematic representation of the role of p63 proteins in regulating ECM remodelling, cell migration and invasion via Src/FAK/AKT/AP-1 signalling pathway. Scale bar represent 100 μm. *N* = 5 (HPV+ve) and 5 (HPV−ve) tumour sections, mean ± SEM, ***p < 0.001 compared to the normal areas.

Oro-pharyngeal tumour sections were also stained for Src and MMP14. Weak cytoplasmic staining for Src and MMP14 was observed in the basal cells, but was not present in the stratified layers of normal epithelium in the HPV positive and negative tumour sections (Fig. 9A). On the contrary, intense cytoplasmic/membrane localisation of Src and MMP14 was observed in both HPV positive and negative tumour areas and this is quantified in Fig. 9A.

## DISCUSSION

Here, we have shown for the first time that enhanced expression of Src induces invasion in E6/E7-HFK by (a) destabilising focal adhesions leading to potentiated cell migration; and (b) regulating expression and activity of MMP14, a pivotal proteinase, via AKT-dependent activation of AP-1 transcription factor c-Jun to promote ECM remodelling. This is all controlled by p63 regulating expression and/or activities of Src, FAK, AKT, c-Jun and MMP14.

Recent studies analysing RNA-seq and microarray data sets from HPV-positive and negative HNSCC have shown an increase in the levels of several transcription factors including p63 [28, 29]. We observed an increase in p63 expression after the expression of HPV16 E6 and E7 genes in primary HFK. However, the induction of invasive incidents was observed only in rafts established with late passage populations of cells (> 12 passages) suggesting that p63-mediated genomic alterations are required for the oncogenic transformation of epithelial cells. Our recent genome-wide analysis of p63 binding sites in HFK identified many p63 targets, which are involved in several pathophysiological processes including cell differentiation, motility, proliferation and ECM remodelling [8, 9].

The high levels of PTK2 and Src, known regulators of cell migration, alongside significant correlation between Src and p63 in OPSCC tumours, implicates Src signalling in the invasive of E6/E7-HFK. Although the mRNA and protein levels of FAK were similar in the non-invasive and invasive population of HFK, its enhanced autophosphorylation was only observed in the invasive population and may be due to up-regulation and clustering of integrins, which are also known targets of p63 [8, 30]. In addition, p63 appears to regulate the expression of these tyrosine kinases as its depletion decreased levels and inhibited the formation of activated Src-FAK complex, thus impairing cell migration. These results go hand in hand with previous studies on FAK-null mice and Src knockdown cells, which also showed impaired migration in fibroblasts with peripheral reappearance of large focal contacts [10, 11, 17]. Specific inhibition of FAK (PF573228) and Src (dasatinib) activities impaired cell migration and invasion in organotypic cultures, with minimum affects on cell proliferation. Suppression of Src and FAK activities in breast cancer support our data and emphasise the Src-FAK-evoked cell migration is a major event in the biology of invasion [11].

The pronounced expression and activity of MMP1, 2, 9 and 14 in oro-pharyngeal tumours and in the invasive E6/E7-HFK indicates that MMPs have a significant role in invasion. However, MMP14 is up-regulated in both HPV positive and negative tumours suggesting that this pathway is active in both types of cancer. Our knockdown studies in E6/E7-HFK confirm that the MMP14 activity is pivotal in driving invasion. This conclusion is of no surprise since MMP14 is known to activate gelatinases and also acts as a collagenase by digesting collagen-I, II and III to promote invasion [31]. In addition, MMP14 also plays a key role in potentiating cancer cell migration [32, 33]. These results are in agreement with recent findings, which indicate a poor 5-year survival rate in patients with increased MMP14 expression regardless of HPV status and make it a potential target since its selective inhibition blocked tumour growth, invasion and angiogenesis [34, 35].

Since specific inhibitors of Src and FAK were effective in reducing the number of invasions, we hypothesised that the increased Src-FAK activity may regulate expression and/or activity of MMP14. Gene knockdown or inhibition of FAK and Src activities attenuated the mRNA, protein levels and activity of MMP14. While MMP14 levels are similar in early and late passage E6/E7 expressing HFKs, only the latter invade since only the late passage cells have increase Src levels and activity. The importance of this is shown when adenovirus-mediated Src-WT or its constitutively active construct (Src-531) are expressed in early passage E6/E7-HFK, which do not normally invade, enhanced mRNA and protein expression of Src and MMP14 with concomitant increase in their activities resulted in invasion on rafts. These results indicate that activated Src transcriptionally regulates MMP14 levels and also regulate its posttranslational modification and cytoplasmic trafficking, which are paramount to its activity. Recent studies in gastric carcinoma, breast cancer and chondrosarcoma cells exemplify the Src-FAK-mediated regulation of MMPs like MMP9 and MMP13 via activation of AP-1 transcription factors like c-Jun and c-Fos [36, 37]. In addition, active Src is known to directly phosphorylate MMP14 at Tyr^573^ and promote its mono-ubiquitination at Lys^581^, which enhances its cytoplasmic trafficking and increases cellular migration and invasion through collagen-I matrix [38, 39]. In breast cancers, activated Src-FAK signalling enriches the cell surface with active MMP14 to promote metastasis, thus indicating posttranslational modification and membrane localisation are essential in MMP14-mediated invasion [40, 41].

To delineate the Src-FAK-dependent downstream signalling pathways, which may induce the expression of MMP14, we focused on c-Jun, since its expression and phosphorylation transcriptionally regulate MMPs and could be linked with tumour progression [36]. Since p-c-Jun-Ser^73^ levels were higher in the invasive population of E6/E7-HFK, it suggests an enhanced transcriptional activity of AP-1 could induce MMP14 expression. Indeed, ectopic levels of Src-WT or Src-531 in early passage E6/E7-HFK enhanced the p-c-Jun-Ser^73^ levels and up-regulated the mRNA and protein expression of MMP14. In contrast, knockdown of c-Jun depleted the MMP14 expression and abrogated the invasion on rafts. Previously, we had shown the AKT-mediated induction of MMP1 via ETS2-dependent mechanism [22]. Since ETS transcription factors regulate the expression and activity of c-Jun in melanoma cells, AKT signalling may also regulate expression and activity c-Jun [42]. Specific inhibition or depletion of AKT reduced the mRNA and protein levels of c-Jun and MMP14 and supported our hypothesis [43].

Our recent genome wide ChIP-seq analysis [9] and the results presented here, identify MMP14 as target of p63. Hence, we studied the role of p63 in regulating the Src/FAK/AKT/AP-1/MMP14 signalling pathway. Knockdown of p63 in the invasive population attenuated the mRNA and protein expression and/or activities of Src, FAK, AKT, c-Jun and MMP14 indicating that p63 orchestrates the Src-mediated invasion. Since the ectopic levels of Src failed to induce expression/activity of MMP14 in cells depleted of either p63 or c-Jun, it appears that p63 and AP-1 transcription factors synergistically regulate the expression of MMP14 (Fig. 9B) [44].

In summary, p63 drives invasion in E6/E7-HFK by modulating Src-FAK signalling which (a) triggers focal adhesion disassembly leading to cell migration, and (b) evokes expression and activity of MMP14 resulting in enhanced ECM remodelling. The combination of specific inhibitors of Src or FAK with MMP14 can be of therapeutic importance in a cancer that is overdue new treatments.

## MATERIALS AND METHODS

### Cell culture

Primary human foreskin keratinocytes (HFK) expressing either control vector (pBabe) or HPV16 E6/E7 genes were cultured in Epilife medium (Life technologies, UK) to generate early (<10 passage) and late passage (> 12 passage) cells [45]. The 3D-organotypic rafts were maintained in the E-medium for 14 days as previously described [46]. The late passage HFK cultured as monolayer or on the rafts were treated with either PF573228 (Tocris, UK) or dasatinib in their respective medium. Each organotypic raft was sliced in three parts (approximately 1–1.5 cm each) before embedding in paraffin and sectioned to generate three equal sized raft sections. These sections were used for H&E staining and immunofluorescence experiments. The invasive incidents were quantified using the three H&E stained sections from each raft and represented as invasive incidents/cm. This was carried out for three independent rafts generated form different HFKs cultures and statistics were performed using the number of invasive incidents/cm recorded from three independent experiments.

### Site-directed mutagenesis and adenovirus generation

The human wild type (WT)-Src construct (pDONR223-Src) (Addgene) was used to generate constitutively active (Src-531) and kinase dead (Src-KD) constructs by introducing STOP codon after Tyr^530^ and replacing lysine by methionine (K298M), respectively, by QuikChange II-Site-directed Mutagenesis (Stratagene, UK) according to the manufacturer's guidelines [47]. After mutations were confirmed by Bigdye terminator sequencing reaction (Life technologies, UK), 5 μg of individual constructs were recombined with the adenoviral pAd/CMV/V5-DEST Gateway vector (Life technologies, UK) using Gateway-LR Clonase-II enzyme mix (Life technologies, UK) according to the manufacturer's guidelines. All recombinant adenovirus were generated using 293T cells and were purified and titrated as previously described [48].

### Retroviral constructs and stable knockdown

The stable knockdown by shRNA molecules in late passage E6/E7-HFK was achieved by retroviral transduction of pSuper-retro-neo constructs (scram, p63). The retroviral shRNA constructs were produced in 293T cells by using the phoenix system as previously described [22, 49].

### Transient knockdown

50 nM of either non-specific (Scram) or specific siRNA targeting total FAK, Src, AKT, c-Jun, p63 and MMP14 (Supplemental Fig. 5) were used to knockdown the target gene and protein expression in E6/E7-HFK as previously described [23].

### Cell migration assay

The HFK seeded on collagen-I coated coverslips were treated with mitomycin C (2 μg/mL) for 2 hours before introducing a scratch wound with a yellow pipette tip. Three bright field images were taken immediately after the scratch-wound was generated (tim*e* = 0 hr) and again in the same field after 20 hours. The rate of cell migration was calculated by the formula: (wound area at *t* = 0 hr - wound area at *t* = 20 hr)/20 for the three areas of each scratch wound and represented as μm^2^/hr. This was carried out in three independent experiments of scratch-wounds generated from three different HFKs cultures and statistics were performed using data from all the three independent experiments.

### RNA extraction and quantitative real time-polymerase chain reaction (qRT-PCR)

RNA extraction was carried out using Trizol (Roche, US) according to the manufacturer's instructions. For qRT-PCR, RNA was reverse transcribed to cDNA by transcriptor first strand cDNA synthesis kit (Roche, US) and the relative mRNA levels of genes were measured as previously described [23].

### Western blotting

The protein levels of FAK, p63 (4A4), MMP1, MMP2, BrdU (Santacruz Biotech), Src, pFAK-Y^395^, MMP14 (Abcam), paxillin (BD biosciences), pPaxillin-Y^118^, pFAK-Y^576/577^, pSrc-Y^496^, pAKT-S^493^, total AKT, p-c-jun-S^73^, c-Jun (Cell Signaling) and β-actin (Sigma-aldrich) were detected in whole cell lysates by immunoblotting as previously described [50].

### Immunofluorescence

To study the cellular localisation of proteins, monolayer of HFK was fixed (4% paraformaldehyde), permeabilised and exposed to primary and species-specific secondary antibodies as previously described [50]. To study proliferation, the rafts were pulsed for 16 hours with BrdU prior to sectioning. The antigens in the raft and oro-pharyngeal tumour sections were retrieved by heat induced epitope retrieval methods (tris buffer, citrate buffer) and the proteins were visualised through 20 × magnification as previously described [49]. The intensities of staining were quantified by calculating the Q-score for proteins in normal and tumour areas. Q-score = Sum of (degree of intensity (-, +, ++, +++) × (% of tissue section))

### Gelatin zymography

To measure the extracellular activities of MMP2 and 9, the conditioned Epilife medium was normalised against the cell number and run on 7.5% SDS-PAGE containing 0.1% gelatin. The enzyme activities were observed by MMP-mediated digestion of gelatin as previously described [51].

### Cell viability

Small aliquots of cell suspension were mixed with equal volume of 0.1% trypan blue for 5 minutes. The mixture was pipetted on haemacytometer, and percentage of viable cells was calculated by counting unstained cells per 100 cells.

### Microarray analysis

The HNSCC microarray analysis was carried out as previously described [23]. Briefly, differential gene expression analysis of Robust Multi-array Average normalized data [24] was used to compare HPV-positive and -negative tumours with normal tissue using a three-way ANOVA model incorporating tumour/normal, site and HPV status.

### Statistics

Results were presented as mean ± SEM. Statistical analysis was performed by using IBM SPSS 20.0 software and comparing the mean by student's *t*-test and one-way analysis of variance (ANOVA) followed by Dunnet's post hoc analysis. The *P* < 0.05 was considered to be significant in all the experiment.

## SUPPLEMENTARY MATERIALS FIGURES AND TABLES


