# Supplementary material for: p63 drives invasion in keratinocytes expressing HPV16 E6/E7 genes through regulation of Src-FAK signalling
Source: Oncotarget. 2015 May 7;8(10):16202–19. doi: 10.18632/oncotarget.3892 (PMC5369957; doi:10.18632/oncotarget.3892)
Supplement: Supplementary file 1 [file oncotarget-08-16202-s001.pdf]

SUPPLEMENTARY FIGURES

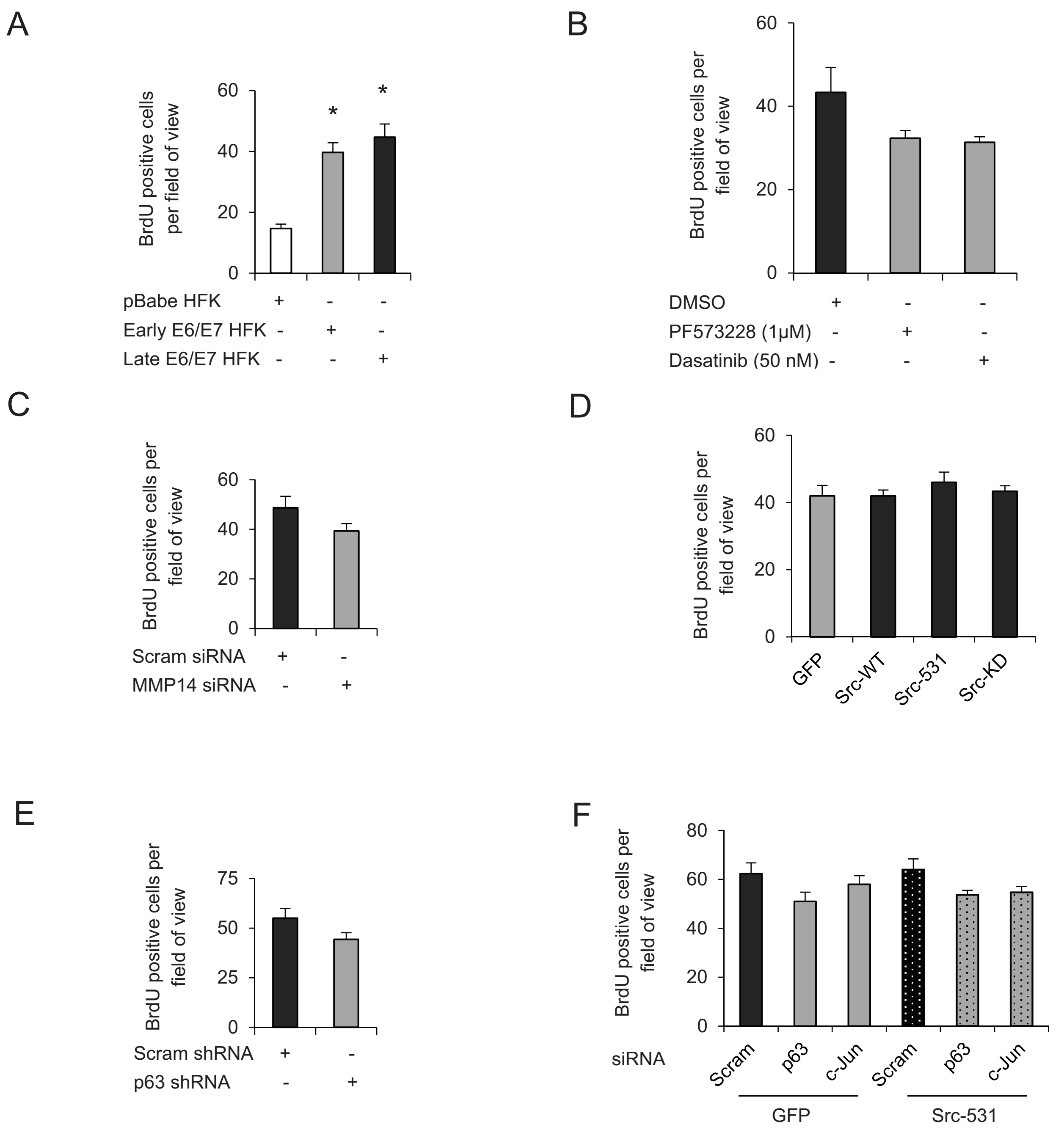

Supplementary Figure 1: Quantification of BrdU positive cells per field of view to address the rate of cell proliferation on the rafts established with A. human foreskin keratinocytes (HFK) expressing pBabe (control), early passage and late passage HFK expressing HPV16 E6/E7 genes. B. late passage E6/E7-HFK treated with specific inhibitors of FAK (PF573228) and Src (dasatinib) activities. **C.** late passage E6/E7-HFK transfected with non-specific (Scram) or MMP14 siRNA (50 nM). **D.** early passage E6/E7-HFK expressing GFP, wild type Src (Src-WT), constitutively active (Src-531) or kinase dead (Src-KD) constructs. **E.** late passage E6/E7-HFK expressing either non-specific (Scram shRNA) or stable p63 knockdown (p63 shRNA). and **F.** late passage E6/E7-HFK depleted with p63 or c-Jun by siRNA knockdown, and expressing GFP or constitutively active Src (Src-531) constructs. $N = 3$ independent experiments, mean ± SEM. $*p < 0.05$ compared to the control.

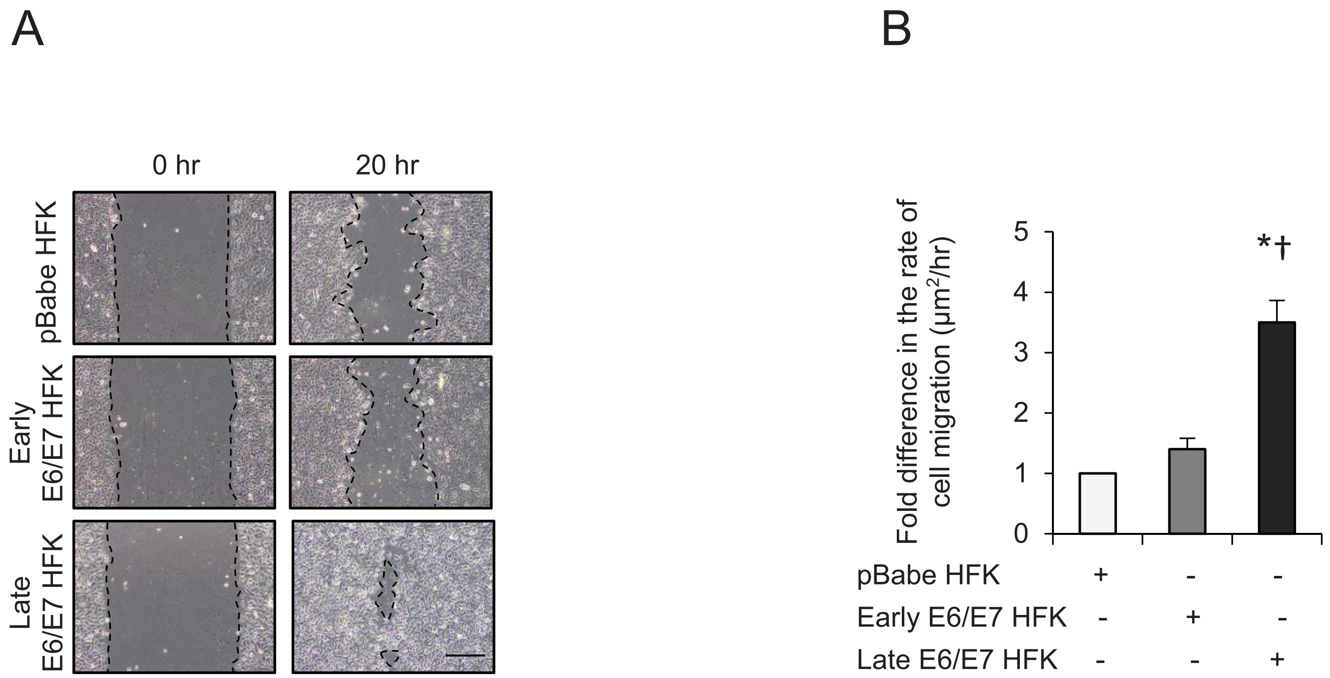

Supplementary Figure 2: Enhanced rate of cell migration in late passage human foreskin keratinocytes (HFK) expressing HPV16 E6/E7 genes. A. Representative phase contrast images of scratch wound assay measuring the rate of cell migration after 20hr in normal HFK expressing pBabe (control), early passage and late passage E6/E7-HFK, which was **B.** quantified and represented as μm^2/hr. Scale bar represent 100 μm. $N = 3$ independent experiments, mean ± SEM, $*p < 0.05$ compared to the control, $^{\dagger}p < 0.05$ compared to early passage E6/E7-HFK.

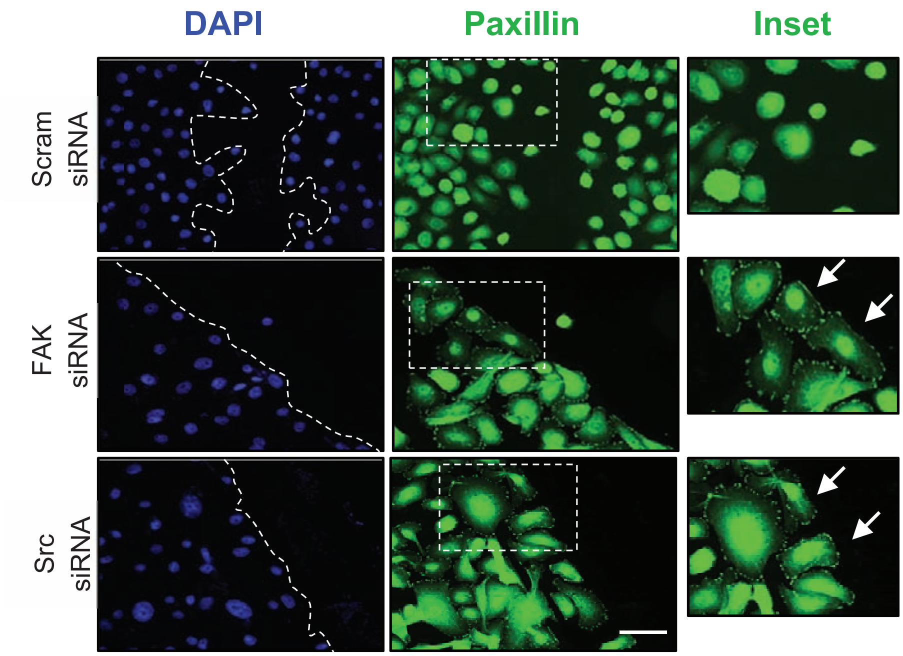

Supplementary Figure 3: Src or focal adhesion kinase (FAK) knockdown impairs the cell migration. **A.** Immunofluorescence detection of paxillin in late passage human foreskin keratinocytes (HFK) expressing HPV16 E6/E7 genes after transient knockdown by Scram (control), FAK and Src siRNA molecules, where arrows represent the peripheral re-localisation of paxillin in the FAK and Src knockdown population (Inset). Scale bar represent 100 µm. $N = 3$ independent experiments.

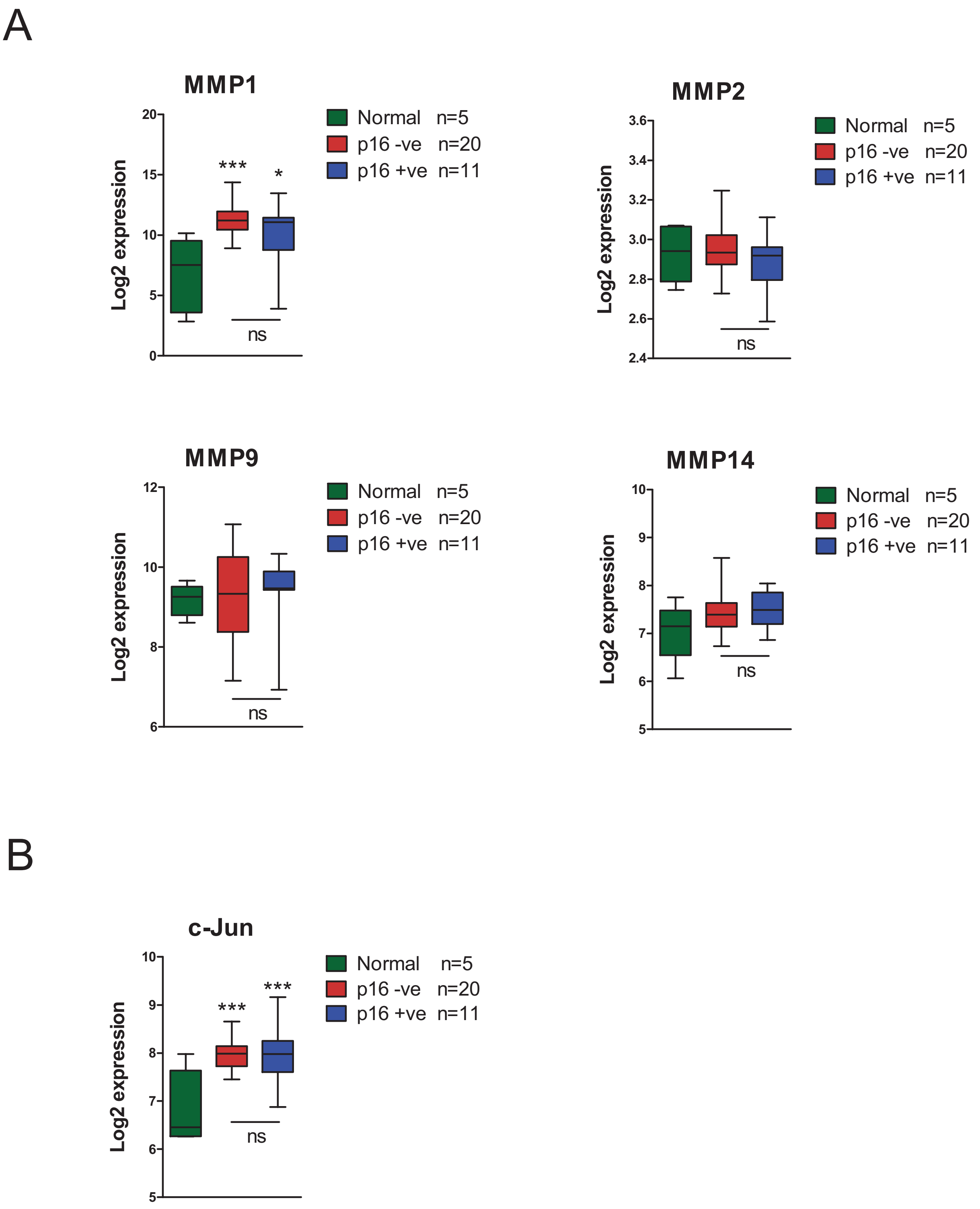

Supplementary Figure 4: Microarray data analysis of A. MMP1, 2, 9, 14 and B. c-Jun levels in normal tissue and HPV-negative (p16 −ve) and HPV-positive (p16 +ve) tumours from oropharyngeal squamous cell carcinomas. Mean ± SEM, ***$p < 0.001$ compared to normal, *$p < 0.05$ compared to normal, ns: not significant.

siRNA Oligonucleotides

Scram:	GCAGCACGACUUCUUCAAG
p63-1:	UCACGACAGUCUUGUACAA
p63-2:	GGACAGUACAAAGAACGGU
Src:	CTCCATGTGCGTCCATATTTA
FAK:	GCATGTGGCCTGCTATGGA
c-Jun-1:	GCAAACCTCAGCAACTTCAACCCAG
c-Jun-2:	GCAAAGATGGAAACGACCTTCTATG
MMP14:	CAGCGATGAAGTCTTCACTTA
Total AKT:	GGACGGGCACATTAAGATCTT

shRNA Oligonucleotides

Total-p63 forward:
GATCCCCAACCATGAGCTGAGCCGTGAATTTTCAAGAGAAATTCACGGCTCAGCTCATGGTTTTTTTG
GAAA

Total-p63 reverse:
GATCCCCAACCATGAGCTGAGCCGTGAATTTTCAAGAGAAATTCACGGCTCAGCTCATGGTTTTTTTG
GAAA

Supplementary Figure 5: siRNA and shRNA oligonucleotide sequences of the targeted genes.
